# Analysis of Infrared Signature Variation and Robust Filter-Based Supersonic Target Detection

**DOI:** 10.1155/2014/140930

**Published:** 2014-02-11

**Authors:** Sungho Kim, Sun-Gu Sun, Kyung-Tae Kim

**Affiliations:** ^1^Yeungnam University, Gyeongsan-si, Gyeongsangbuk-do 712-749, Republic of Korea; ^2^Agency for Defense Development, 111 Sunam-dong, Daejeon 305-152, Republic of Korea; ^3^Electromagnetic Technology Laboratory, POSTECH, Pohang 790-784, Republic of Korea

## Abstract

The difficulty of small infrared target detection originates from the variations of infrared signatures. This paper presents the fundamental physics of infrared target variations and reports the results of variation analysis of infrared images acquired using a long wave infrared camera over a 24-hour period for different types of backgrounds. The detection parameters, such as signal-to-clutter ratio were compared according to the recording time, temperature and humidity. Through variation analysis, robust target detection methodologies are derived by controlling thresholds and designing a temporal contrast filter to achieve high detection rate and low false alarm rate. Experimental results validate the robustness of the proposed scheme by applying it to the synthetic and real infrared sequences.

## 1. Introduction

**Figure 1:**
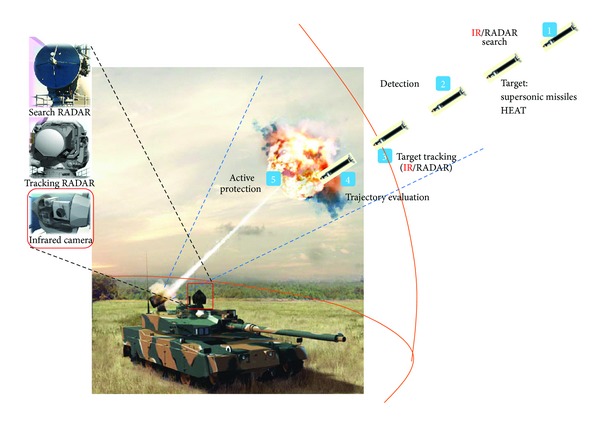
IR-based target detection and tracking in active protection system (APS).

Small infrared target detection is important for a range of military applications, such as infrared search and track (IRST) and active protection system (APS). In particular, an APS is a system designed to protect tanks from guided missile or rocket attack by a physical counterattack, as shown in Figure 1. An antitank missile, such as high explosive antitank (HEAT), should be detected at a distance of at least 1 km and tracked for active protection using RADAR and infrared (IR) cameras. Although RADAR and IR can complement each other, this study focused on the IR sensor-based approach because it can provide a high resolution angle of arrival (AOA) and detect high temperature targets.

IR sensors are inherently passive systems and do not have all weather capability. In addition, IR images show severe variations according to background, time, temperature, and humidity, which makes the target detection difficult. The use of adaptive IR sensor management techniques can enhance the target detection performance. On the other hand, few studies have analyzed the IR variations in terms of small target detection using the data collected over a 24-hour period. In 2006, Jacobs summarized the basics of radiation, atmospheric parameters, and infrared signature characterization [1]. He measured the thermal variations in various environments. Recently, the TNO research team characterized small surface targets in coastal environments [2–4]. In 2007, the TNO team introduced the measurement environment and examined the target contrast and contrast features of a number of small sea surface vessels [2]. The analysis revealed a variation in contrast due to changes in solar illumination, temperature cooling by wind and sun-glint. In 2008, the team analyzed the variations in the vertical profile radiance and clutter in a coastal environment [3]. Based on the analysis, a Maritime Infrared Background Simulator (MIBS) background simulation was performed under these measurement conditions. They can predict clutter in coastal background accurately. In 2009, they extended the IR-based analysis to visible cameras, hyperspectral cameras, and polarization filters to validate the contrast and clutter model [4].

The first contribution of this paper is IR variation analysis in terms of small target detection. The second contribution is the acquisition of 24-hour IR data in winter and spring. The third contribution is variation analysis for different backgrounds. The fourth contribution is the proposition of robust small target detection based on the variation analysis.  Section 2 characterize the infrared signature and presents the sources of infrared variations.  Section 3 explains the target detection parameters and variation measurement results including the details of the IR variability in different background, time, temperature, and humidity.  Section 4 proposes small target detection methods to overcome the target signature variations.  Section 5 presents the experimental results for synthetic and real test sequences.  Section 6 discusses the analysis results and concludes the paper.

## 2. Characterization of Infrared Target Signature and Variation

### 2.1. Physical Modeling of Infrared Target Signature

**Figure 2:**
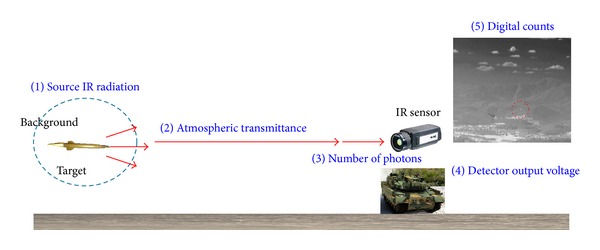
IR target imaging process and sources of IR variations.

In general, IR target images are obtained by the process of IR radiation contrast, attenuation by atmospheric transmittance, photon to voltage conversion in the IR sensor, and analog-to-digital conversion (ADC), as shown in Figure 2. The temperature contrast between the target and background is radiated and propagated in the air. The radiation contrast is attenuated by atmospheric effects, such as absorption, emission, and scattering of H_2_O and CO_2_.

Objects with temperatures higher than 0 K radiate thermal energy, and such energy differences create voltage differences in thermal detectors. Most target detection algorithms use the difference information between target and background energy. Based on such basic radiation theory, we can predict relative target digital signal levels given the information of thermal radiation intensity difference between the target and background by carefully modeling the energy transformation processes. Modeling procedures are as follows. First, we input a thermal radiant intensity difference between the target and background. Second, we calculate atmospheric transmissivity. Third, we calculate the number of photons in front of the sensors. Fourth, we calculate the voltage levels at a detector. Finally, we obtain gray levels or digital counts after analog-to-digital (A/D) converter.

Since the objective of APS is to detect distant targets as early as possible, targets can be regarded as point sources. Therefore, we use the output voltage model in the thermal detector as the following equation [5]:

(1) ΔVpnt=G∫λ1λ2Rd(λ)I(λ,ΔT)A0R2·PVF  ·τO(λ)·Tatm(λ)dλ,

where *I*(*λ*, Δ*T*) [watt/sr *μ*m] denotes radiant intensity at wavelength (*λ*) when the temperature difference is Δ*T*. *R* [m] represents the distance between the IR camera and a target. These two parameters should be provided by users. Δ*V*_pnt_ means voltage difference produced by target region and background region in a thermal detector. *T*_atm_(*λ*) denotes atmospheric transmissivity that is defined as a ratio (the fraction or percent of a particular frequency or wavelength of electromagnetic radiation that passes through a medium without being absorbed or reflected). *A*_0_ [m^2^] represents the area of entrance aperture of the IR camera. *τ*_*O*_(*λ*) represents the optical transmissivity of the IR camera, which considers the mirrors and lenses. Ideally, the thermal energy of a distant point target should be gathered in a pixel of a detector. However, thermal energy of a point target is dispersed (blurred) due to the diffraction and aberration of the optical system. PVF (point visibility factor) can model such phenomenon quantitatively. So, PVF is defined as the ratio of center pixel energy over total target energy. *λ*_1_, *λ*_2_ represent operation ranges, lower limit, and upper limit of thermal detector. *R*_*d*_(*λ*) denotes detector responsivity versus wavelength. If the responsivity is available, then we can estimate the output voltage given infrared intensity. *G* denotes the detector gain. We usually use radiant intensity Δ*I* [watt/*sr*⁡] by integrating *I*(*λ*, Δ*T*) with *λ*_1_~*λ*_2_. Furthermore, if we use average transmissivity, optics transmissivity, and detector responsivity within wavelength, then (1) is simplified as the following equation:

(2) ΔVpnt=ΔI×Tatm×A0R2×τO×PVF×Rd×G.

Given radiant intensity (Δ*I*) of target-background, the total number of photons is calculated by dividing the radiant intensity by energy per photon as in the following equation:

(3) $$\begin{array}{c} \Delta E=\frac{\Delta I}{hc/\lambda_{\text{center}}}, \end{array}$$

where *h* = 6.626 × 10^−34^ [Js] denotes Plank's constant, *c* = 3 × 10^8^ [m/s] denotes the speed of light, and *λ*_center_ = (*λ*_1_ + *λ*_2_)/2 means center wavelength.

The responsivity and gain are determined by quantum efficiency (*η*_*Q*_), electron charge (*q* [C]), integration time (*T*_*i*_[*s*]), dark current (*I*_dark_), and equivalent capacitance (*C*_eq_) in the readout integrated circuit. If we use this information, then (2) is rewritten as the following equation:

(4) ΔVpnt=(ΔE×Tatm×A0R2×τO×ηQ×q+Idark)×PVF×1Ceq.

Since the dark current in a cooled thermal detector is so small, it can be removed in the above. In addition, the estimated PVF is reflected by Gaussian filtering in the image domain. So, the final form for point source is simplified as the following equation:

(5) $$\begin{array}{c} \Delta V_{\text{pnt}}=\left(\Delta E\times T_{\text{atm}}\times \frac{A_{0}}{R^{2}}\times \tau_{O}\times \eta_{Q}\times q\right)\times \frac{1}{C_{\text{eq}}}. \end{array}$$

The atmospheric transmissivity is calculated using MODerate resolution atmospheric TRANsmission (MODTRAN) and Beer's law [6] according to the target distance. In Beer's law, atmospheric transmittance is defined as *T*_atm_ = *e* ^−*γR*^, where *τ* denotes attenuation coefficient (km^−1^). If the target distance (*R*) is larger than 20 km then Beer's law is used. Otherwise, we use MODTRAN to estimate atmospheric transmissivity.

Let us assume that a target of Δ*I* [watt/sr] is at distance *R* [m]. If the projected target size is smaller than 1 pixel, then we use (5) to calculate the difference voltage output. Digital value of difference voltage is obtained as (6) by considering the bit resolution (*m* [bit]) in the A/D converter and voltage dynamic range (Δ*V*_dynamic_):

(6) $$\begin{array}{c} \Delta D=\frac{2^{m}}{\Delta V_{\text{dynamic}}}\cdot \Delta V. \end{array}$$

### 2.2. Sources of Signature Variations

The radiation contrast between the target and background can be used to target detection. However, it is challenging problem due to the dynamic behavior of the radiation contrast (Δ*E*) and atmospheric transmissivity (*T*_atm_). According to Jacobs's analysis [1], IR signature variations are generated by the target conditions, environmental variations, and material properties. The target conditions included exhaust grid/gases, crew compartment heating/cooling, power generator, material properties, camouflage, target location, and orientation. Because the targets (HEAT) in APS are small (length: 1 m, diameter: 0.1 m) and only incoming targets are considered, the variations caused by the target conditions were not considered in the present study. The environmental variations include the induced weather (sun, clouds, rain, and snow), atmospheric effects on transmission, and the geographical location. In this study, IR variations caused by 24-hour weather in winter and spring were considered. The material properties can be another source of IR variations. Different materials in different backgrounds exhibit different IR signatures. For example, there are trees in remote mountains, concrete buildings in urban areas, soil and grass in near fields, and air/cloud in the sky. The material properties are related to the radiation contrast between target and background. Environmental weather conditions are related to the atmospheric transmissivity.

## 3. Variation Analysis of Infrared Target Signature

### 3.1. Basic Parameters of Small Target Detection

**Figure 3:**
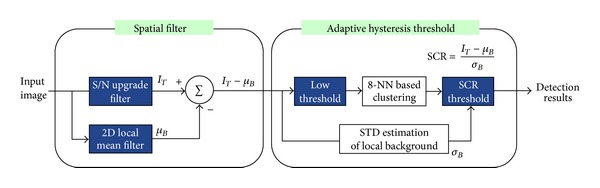
Spatial filter and adaptive hysteresis threshold-based small infrared target detection system.

In the infrared small target detection community, background subtraction-based approaches are well established and embedded in military systems. In 2011, Kim proposed a modified mean subtraction filter (M-MSF) and a hysteresis threshold-based target detection method [7]. As shown in Figure 3, an input image (*I*(*x*, *y*)) is pre filtered using Gaussian coefficients (*G*_3×3_(*x*, *y*)) to enhance the target signal and reduce the level of thermal noise according to the following equation:

(7) $$\begin{array}{c} I_{T}\left(x,y\right)=I\left(x,y\right)*G_{3\times 3}\left(x,y\right). \end{array}$$

The signal-to-clutter ratio (SCR) is defined as (max target signal − background intensity)/(standard deviation of background). Simultaneously, the background image (*μ*_*B*_(*x*, *y*)) is estimated using a 11 × 11 moving average kernel (MA_11×11_(*x*, *y*)) as the following equation:

(8) $$\begin{array}{c} \mu_{B}\left(x,y\right)=I\left(x,y\right)*\text{M}{\text{A}}_{11\times 11}\left(x,y\right). \end{array}$$

The pre-filtered image is subtracted from the background image, which produces a target-background contrast image (*I*_contrast_(*x*, *y*)), as expressed in (9). The modified mean subtraction filter (M-MSF) can upgrade the conventional mean subtraction filter (MSF) in terms of false detection:

(9) $$\begin{array}{c} I_{\text{contrast}}\left(x,y\right)=I_{T}\left(x,y\right)-\mu_{B}\left(x,y\right). \end{array}$$

**Figure 4:**
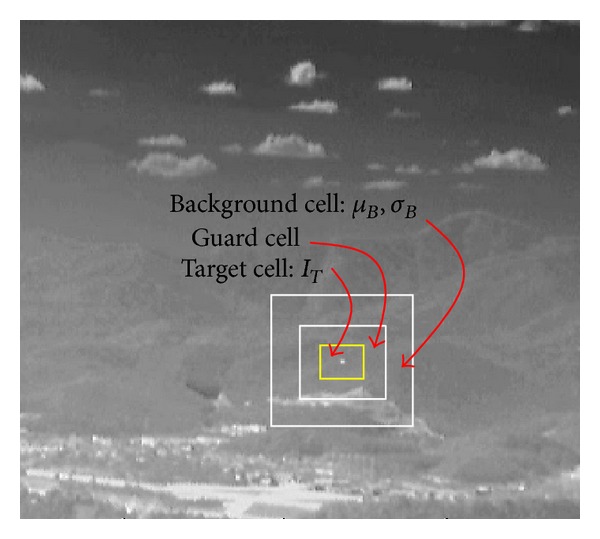
SCR estimation, target cell, guard cell, and background cell are selected using the clustering information.

The last step of small target detection is how to determine which pixels correspond to the target pixels. Kim proposed an adaptive hysteresis thresholding method, as shown in Figure 3. A contrast threshold (*k*_1_) is selected to be as low as possible to locate the candidate target region. The 8-nearest neighbor (8-NN) based clustering method is then used to group the detected pixels. The SCR threshold (*k*_2_) is selected properly to meet the detection probability and false alarm rate, as expressed in (10). A probing region is declared as a target if

(10) $$\begin{array}{c} \text{SCR}\left(x,y\right)=\frac{I_{T}\left(x,y\right)-\mu_{B}\left(x,y\right)}{\sigma_{B}\left(x,y\right)}>k_{2}, \end{array}$$

where  *μ*_*B*_ and *σ*_*B*_ represent the average and standard deviation of the background region, respectively. *k*_2_ denotes the user defined parameter used in the control detection rate and false alarm rate. A probing region is divided into the target cell, guard cell, and background cell, according to the results of contrast thresholding and clustering, as shown in Figure 4. Therefore, the key parameters of small target detection are the SCR-related terms, such as the average background intensity (*μ*_*B*_), target intensity (*I*_*T*_), and standard deviation of the background (*σ*_*B*_). In the SCR computation, the target-background contrast parameter (*I*_*T*_ − *μ*_*B*_) can be derived from the key parameters, which should be analyzed according to the IR variations.

### 3.2. Acquisition of Infrared Images

*Measurement Devices. *The objective of this study was to evaluate the small target detection parameters (*I*_*T*_, *μ*_*B*_, *I*_*T*_ − *μ*_B_, *σ*_*B*_) over a 24-hour period for a range of backgrounds, such as the remote mountain, building, near field, and sky. As shown in Table 1, a LWIR camera, CCD camera, DSLR camera, thermal target, and IR thermometer were used to record the target and environmental information. The FLIR Tau302 can record digital IR data with a 14-bit resolution. The SONY NEX-VG20 can record visible data with HD video resolution. The 5D Mark II was used to record the overall experimental status. The BMH-30 is a thermal target to simulate the plume of an antitank missile. The normal temperature of the target is approximately 450°C. The DT-8865 can measure the temperature of the targets and backgrounds with the range of −50 to 1000°C.

**Figure 5:**
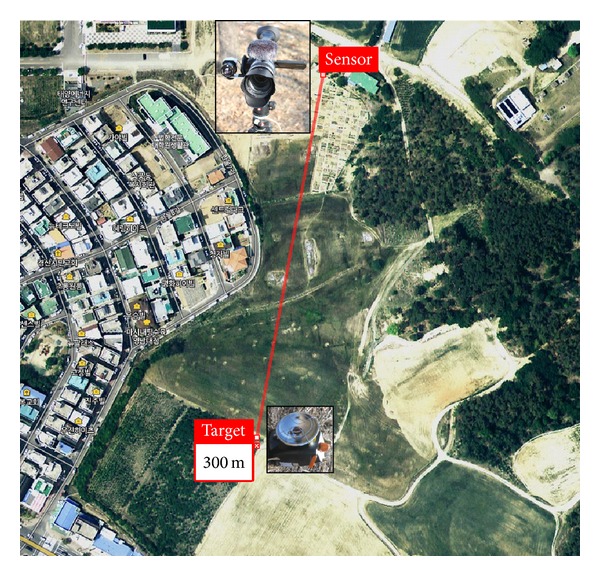
Overview of the target and sensor positions.

*Location and Meteorological Data.* In APS, the incoming antitank missiles should be detected at a distance of at least 1 km and then tracked for the following hard killing process. Because the focus of this study was to analyze the effects of the day/night changes on the SCR parameters for different backgrounds, a small region was selected within the campus, as shown in Figure 5. The distance between sensors and a target is around 300 m. The sensing point is selected carefully to include a variety of backgrounds, such as remote mountains, buildings, near field, and sky. During the recording, the meteorological measurement data from Korea Meteorological Administration web site (KMA, http://web.kma.go.kr) was also checked, as shown in Table 2 (winter) and Table 3 (spring). The tables consist of the recording time, overall weather, visibility, cloud, temperature, and relative humidity. In winter, the overall weather was clear with a temperature and humidity range of −3.0°C~4.8°C and the humidity range of 14%~66%. In spring, the overall weather was cloudy with a temperature and humidity range of 5.6°C~15.4°C and the humidity range of 27%~75%.

**Figure 6:**
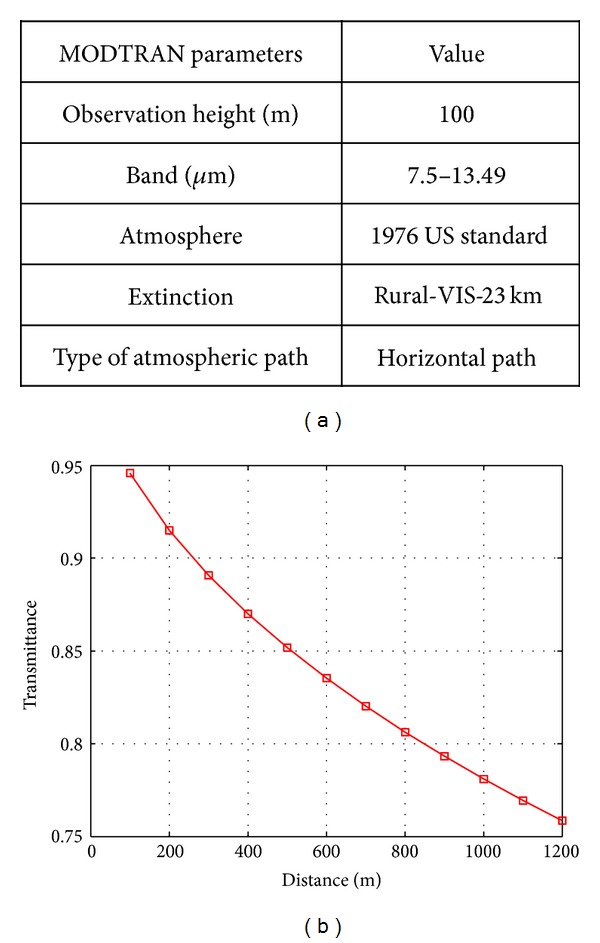
MODTRAN simulation of the recording environment: (a) selected MODTRAN parameters, (b) transmittance versus distance.

Based on the measured weather, the atmospheric transmittance can be simulated using the MODTRAN (MODerate resolution atmospheric TRANsmission, http://modtran5.com/) program designed to model the atmospheric propagation of electromagnetic radiation for the 0.2 to 100 *μ*m spectral range. The simulation parameters were selected, as shown in Figure 6(a).  Figure 6(b) shows the corresponding transmittance according to distance. The transmittance was evaluated from 100 m to 1200 m at 100 m intervals because small targets should be detected and tracked at 1200 m. The transmittance at 1200 m was 0.76 and increases to 0.95 at 100 m. Note that the transmittance was quite high, so the signal attenuation could be negligible due to the atmosphere.

**Figure 7:**
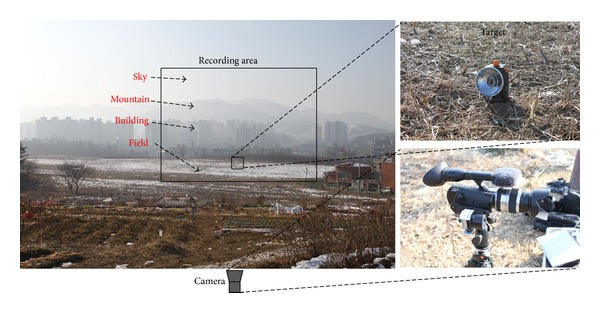
Recording area and locations of the target and cameras.

**Figure 8:**
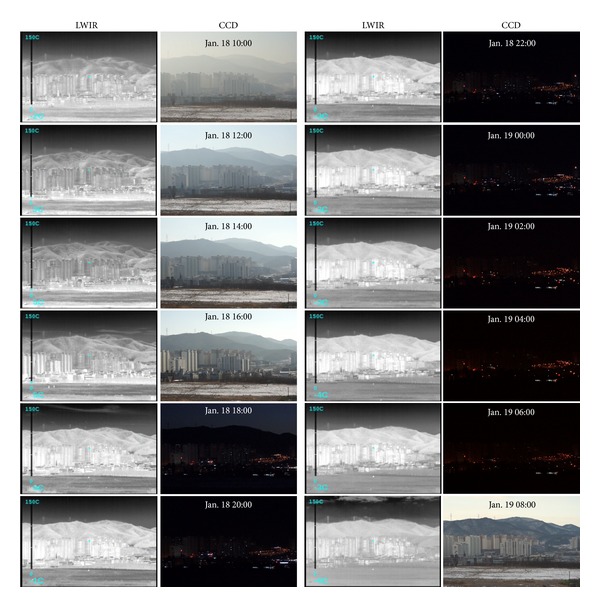
Partial examples of the acquired LWIR and CCD images over 24-hour period in Winter.

**Figure 9:**
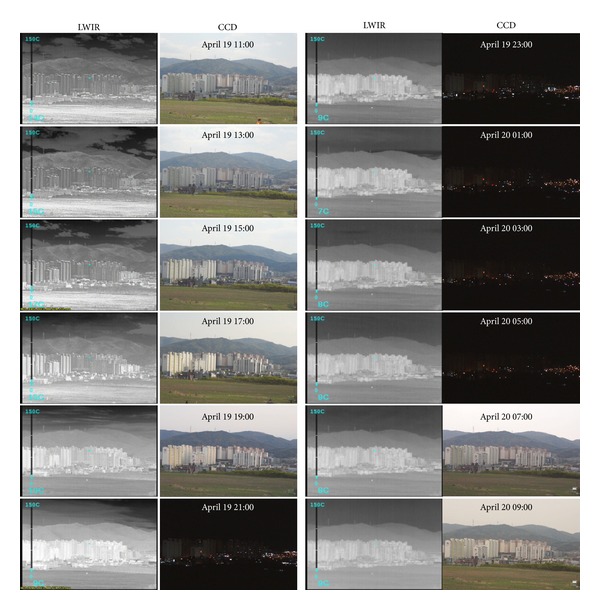
Partial examples of the acquired LWIR and CCD images over 24-hour period in spring.

*Examples of Acquired Images.* The recording area was selected to include sky, mountain, building, and field backgrounds, as shown in Figure 7, where the locations of a target and cameras are indicated. Over a 24-hour period, a pair of LWIR and CCD images was recorded at 1-hour intervals. In LWIR, both digital 14-bit data and contrast enhanced image were acquired for variability analysis. Figures 8 and 9 give partial examples of a 24-hour recording in winter and spring, respectively. The scene temperatures are indicated in the LWIR images and the recording times are displayed on the CCD images.

### 3.3. Variability Analysis of Infrared Images

**Figure 10:**
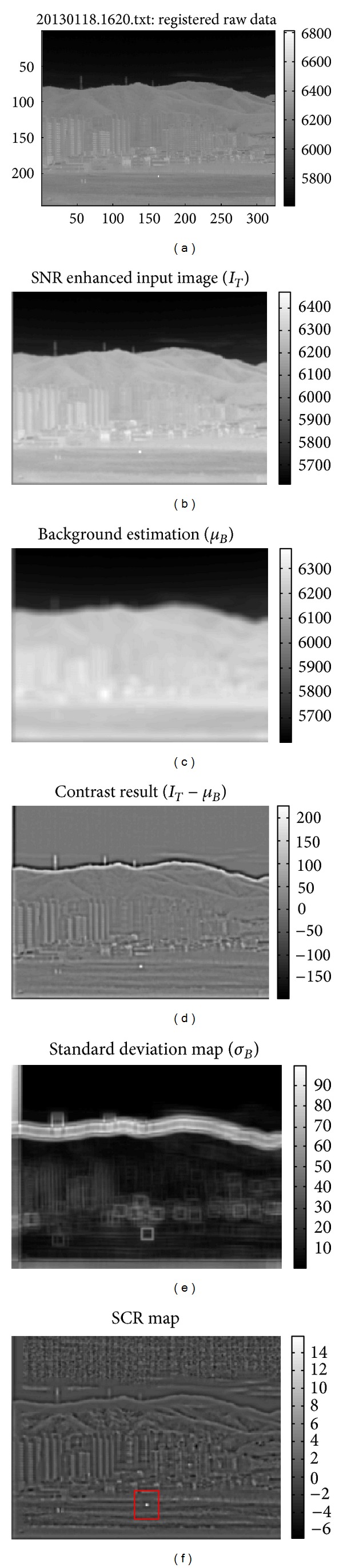
SCR computation flow: (a) registered raw input data (14 bits), (b) SNR enhanced input image, (c) background estimation result, (d) contrast result, (e) standard deviation estimation, and (f) computed SCR map.

*Analysis Factors*. The key parameters in small target detection are the pre-filtered input image (*I*_*T*_), estimated background image (*μ*_*B*_), standard deviation map (*μ*_*B*_). The contrast data and SCR values can be derived based on these three key parameters.  Figure 10 shows the flow of the SCR computation of a test image. The test image was obtained from 14-bit raw data and the bright spot represents a gas heater (Figure 10(a)). SNR enhanced image can be obtained by applying a matched filter to the input image (Figure 10(b)). The background image can be estimated using a local mean filter with a 11 × 11 moving average kernel (Figure 10(c)). A contrast image can be obtained by subtracting the estimated background image from the pre-filtered input image (Figure 10(d)). A standard deviation map was estimated from the contrast image (Figure 10(e)). The final SCR map was generated using the contrast result and standard deviation map (Figure 10(e)).

**Figure 11:**
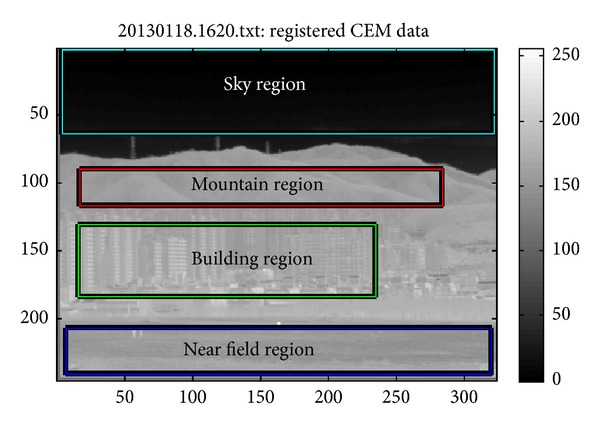
Analysis regions indicated on the image.

The SCR-related parameters should be analyzed on different backgrounds because antitank missiles can exist anywhere. The backgrounds were classified as natural (sky, remote mountain, near field) and artificial background (man-made buildings).  Figure 11 shows the corresponding regions indicated by the rectangles. Because this study was interested in the background variation effects on the SCR values, a fixed target signal, such as 7317 obtained by averaging the target intensities, was used. This assumption is reasonable because the target distance is within 1 km and the signal attenuation is negligible. For each pixel position ((*i*, *j*)), the mean background intensity (*μ*_*B*_(*i*, *j*)), standard deviation (*σ*_*B*_(*i*, *j*)), and SCR value (*S*CR(*i*, *j*)) were calculated. A representative value for each region was obtained by averaging the corresponding values.

*Variation Analysis Results.* The effects of the recording time, temperature, and humidity on the SCR parameters were analyzed. From the analysis, the optimal small target detection time, temperature, and humidity conditions were obtained for different backgrounds. In addition, the evaluated data was used to control the detection thresholds to achieve a predefined detection rate.

**Figure 12:**
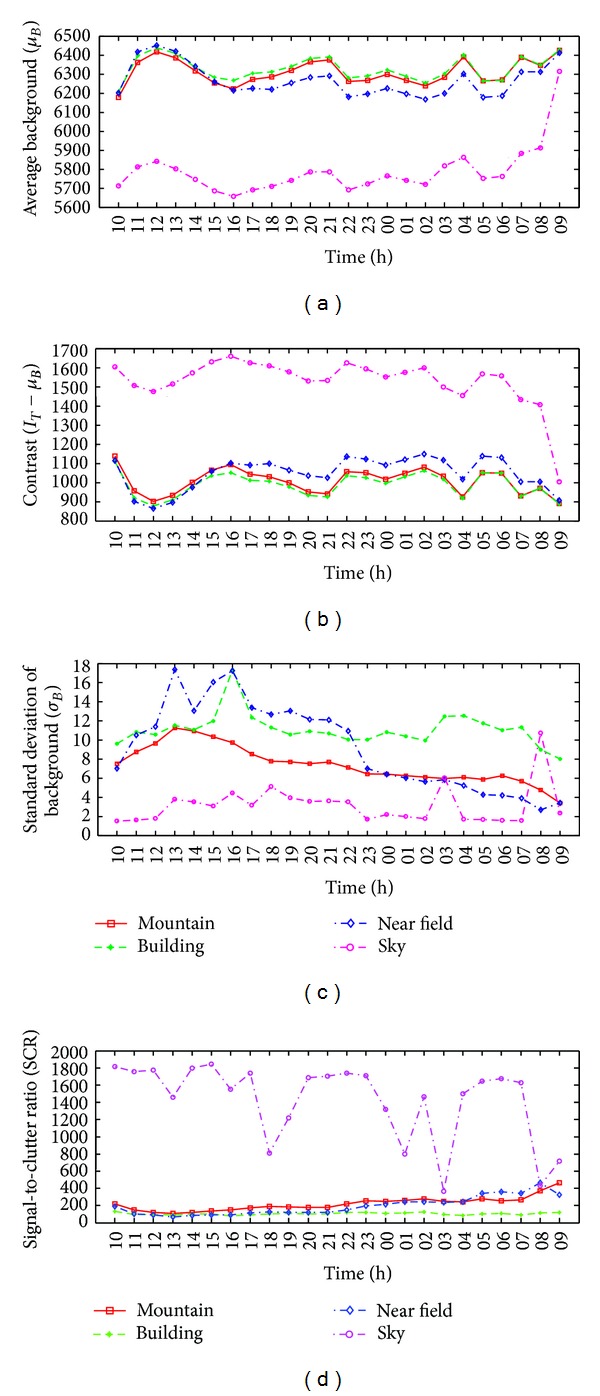
Variation analysis of winter according to the recording time: (a) average background versus time, (b) contrast versus time, (c) standard deviation versus time, and (d) SCR versus time.

In the first inspection, the SCR parameter variations were analyzed according to the recording time. In winter season, the recording time started at 10 a.m. and ended at 9 a.m. on the next day with a 1-hour recording interval.  Figure 12(a) shows the average background intensity (*μ*_*B*_) variations for the four types of backgrounds over a 24-hour period. The background intensities were relatively high during the day and low during the night. Sky background showed very low intensity and increased when a cloud appeared (09 H). Given a fixed target intensity, the contrast data can be calculated as shown in Figure 12(b). The contrast values showed the lowest value at noon and fluctuated during the night. The sky background showed the highest contrast all the time. During the day (10 H–15 H), the contrast magnitudes were as follows: sky > mountain > building > near field. During the night, the order of the changes was sky > near field > mountain > building. The contrast of the building and target decreased at night because humans use energy to warm rooms. The variations in the clutter level can also be checked using the standard deviation of the background, as shown in Figure 12(c). According to the graph, the clutter level increased during the day and decreased during the night. The standard deviation of the sky background almost showed the lowest values but increases when a cloud appeared (03 H, 08 H). The near field showed strong clutter during the day and evening. The building background showed almost constant clutter during the entire day. The clutter level of the mountain background showed a peak at noon and decreased. The final SCR versus time curve can be obtained from the above parameter variations, as shown in Figure 12(d). The mountain and near field background showed increasing SCR values according to the time and the building background showed an almost constant SCR curve. The sky background represents very high SCR values and decreased abruptly when the cloud appeared (03 H, 08 H). As higher SCR values would ensure a higher detection rate, the best operating time can be predicted for each background from the curve.

**Figure 13:**
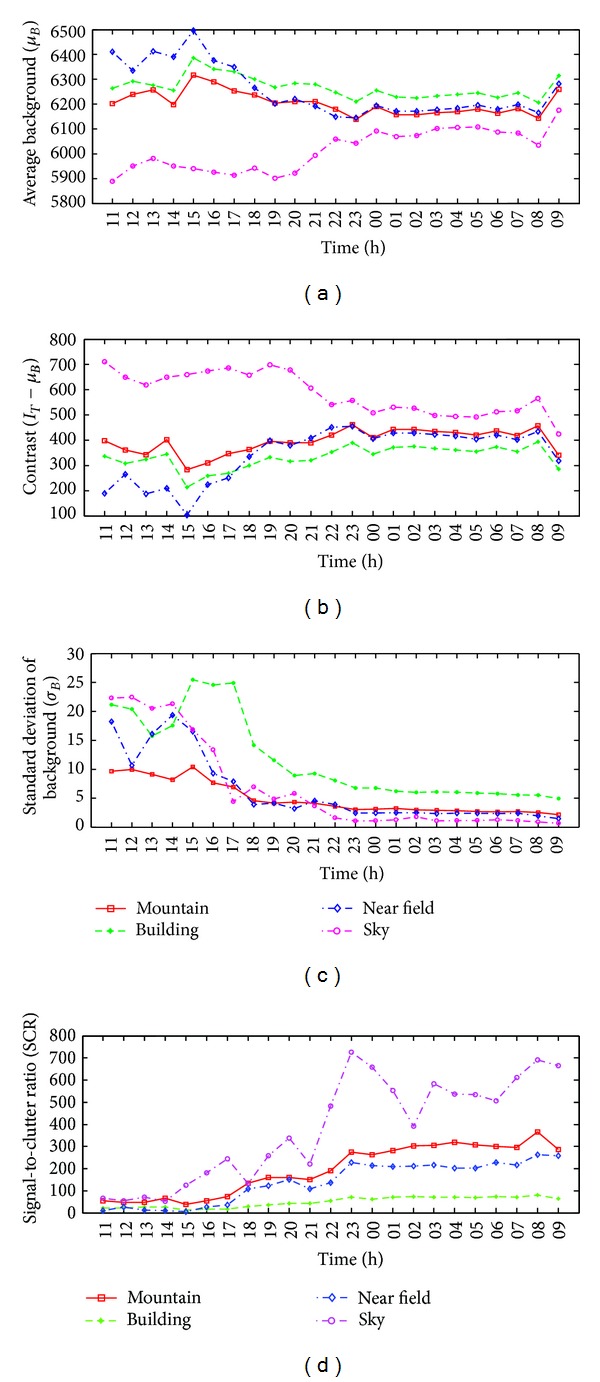
Variation analysis of spring according to the recording time: (a) average background versus time, (b) contrast versus time, (c) standard deviation versus time, and (d) SCR versus time.

In spring season, the recording conditions are the same as the winter season except the starting time (we started at 11 a.m.).  Figure 13(a) shows the average background intensity (*μ*_*B*_) variations for the four types of backgrounds over a 23-hour period. The background intensities were relatively high during the day and low during the night except the sky background. It showed very low intensity and increased when a cloud appeared (after 21 h). Given a fixed target intensity, the contrast data can be calculated as shown in Figure 13(b). The contrast values showed the lowest value at 15 h and remained constant during the night. The sky background showed the highest contrast all the time as the winter case. During the day (11 H–17 H), the contrast magnitudes were as follows: sky > mountain > building > near field. During the night (18 H–08 H), the order of the changes was sky > mountain ≥ near field > building. The contrast of the mountain and near field shows quite similar values and patterns. The variations in the clutter level can also be checked using the standard deviation of the background, as shown in Figure 13(c). According to the graph, the clutter level is high during the day and decreased during the night. The order of clutter level during night was building > mountain > near field > sky. The final SCR versus time curve can be obtained from the above parameter variations, as shown in Figure 13(d). The mountain, near field, and building background showed increasing SCR values according to the time and the sky background represents very high SCR values and fluctuated abruptly according to the level of cloud. Note that the SCR of building background was almost constant in winter and increased in spring during 23 hours.

**Figure 14:**
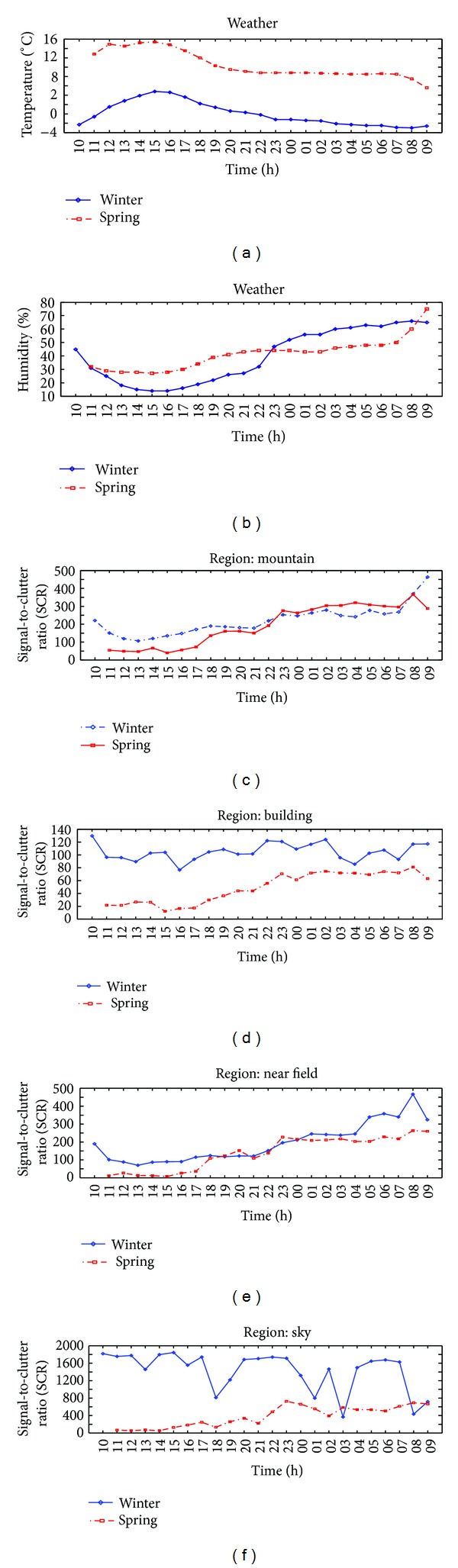
SCR comparison between winter and spring: (a) mountain, (b) building, (c) near field, and (d) sky.

If we compare both data (winter, spring), we can find interesting results as shown in Figure 14. Figures 14(a) and 14(b) represent the temperature and humidity variations according to time. The temperature increased during day and decreased during night. Conversely, the humidity decreased during day and increased during night. The temperature of spring is higher than that of winter and the humidity of spring fluctuates less than that of winter. Figures 14(c)–14(f) compare SCR values between winter and spring for the mountain region, building region, near field region, and sky region, respectively. The SCR curves of the mountain and near field show similar patterns in winter and spring season. However, the SCR curves of the building region show different characteristics: almost constant in winter and increasing pattern in spring. The SCR curves of sky region show quite random according to the level of cloud.

In addition, the SCR parameter variations were analyzed according to the temperature. Because the temperatures were recorded at each recording time, the SCR parameters were reordered according to the temperature. As shown in Figures 14(a) and 14(c)–14(f), SCR values of mountain and near field increased slightly according to the temperature decrease. Those of the sky background do not reveal such phenomena because it is more affected by cloud. Those of the sky background did not reveal such phenomena because it is affected more by cloud. The SCR values of the building background were relatively unaffected by temperature in winter except spring. From above inspection, small targets can be detected better when they are cold (night), particularly in natural backgrounds, such as mountains and near fields.

In the last inspection, the SCR parameter variations were analyzed according to the humidity. Because relative humidity was recorded at each recording time, the SCR parameters could be reordered according to the humidity level. As shown in Figures 14 (b) and 14(c)–14(f), SCR values of mountain and near field increased slightly according to the humidity increase. Those of the sky background did not reveal such phenomena because it is affected more by cloud. The SCR values of the building background were relatively unaffected by the humidity.  Table 4 lists the overall evaluations.

## 4. Small Target Detection Robust to Target Signature Variations

Until now, we discussed the variability of infrared signature in terms of target detection parameter (SCR). A successful small target detection system should work regardless of the signature variations. We can consider two kinds of overcoming approaches such as knowledge-based adaptive thresholding and robust detection filtering method.

### 4.1. Knowledge-Based Thresholding

SCR values change enormously according to recording time, temperature, humidity, season, background type, and so on. If we have a lot of databases, we can make a knowledge-based target detection system using (11) to handle the signature variation. An adaptive threshold (Th) is determined by a function with parameters of time (*θ*_*t*_), temperature (*θ*_*c*_), humidity (*θ*_*h*_), season (*θ*_*s*_), and background type (*θ*_*b*_). In fact, such approach is time consuming and impractical to realize the knowledge database

(11) $$\begin{array}{c} \text{SCR}\left(x,y\right)>\text{Th}\left(\theta_{t},\theta_{c},\theta_{h},\theta_{s},\theta_{b}\right). \end{array}$$

### 4.2. Robust Target Detection Filter

Background subtraction-based small target detection is sensitive to the IR signature variation and generates a lot of false detections by background clutter. However, if we use robust target detection filter, the problem can be mitigated. In the past decades, a variety of approaches have been developed. Among them, the temporal variance filter (TVF) of temporal profile has been used successfully to detect point targets moving at subpixel velocity [8, 9]. Slowly moving cloud clutter can be removed by subtracting the connecting line of the stagnation points (CLSP) [10]. Recently, the CLSP method is approximated for real time processing [11]. In supersonic missile detection with high frame rate camera, a detection algorithm should be simple but powerful performance of detection rate and localization accuracy to cover a wide range of target velocity (subpixel to pixel velocity).

**Figure 15:**
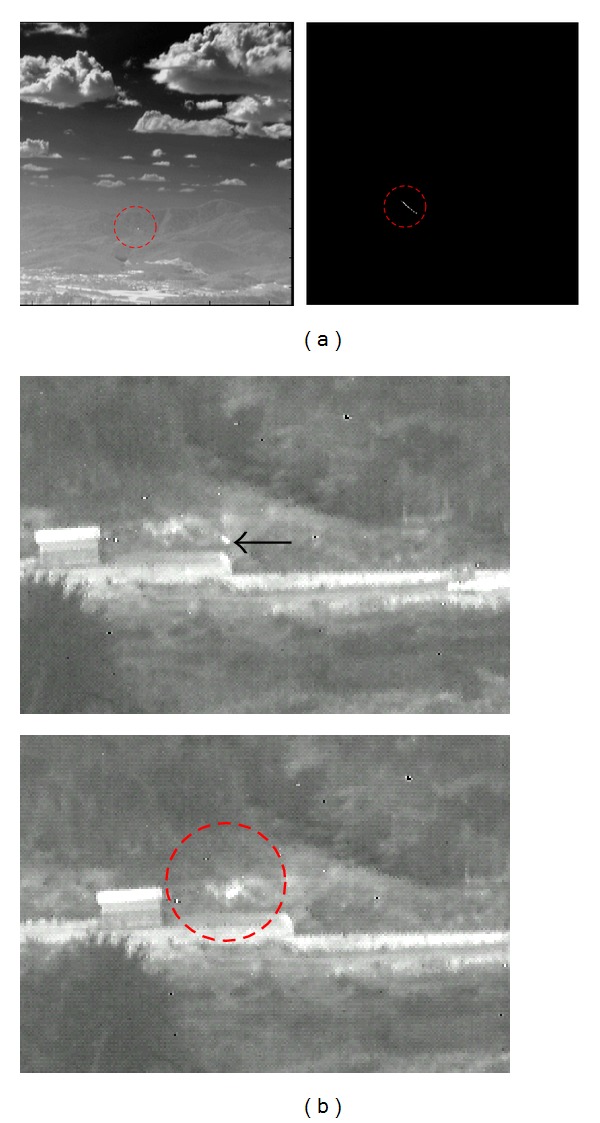
Limitations of TVF-based method: (a) ambiguity of target position and subpixel velocity, (b) target missing by the intersection of TVF and spatial filter.

Because the TVF-based method detects targets based on stripe patterns, it shows high detection performance. However, it has limitations such as the ambiguity of target position and subpixel velocity assumption as shown in Figure 15(a). The ambiguity of target position can be solved by the intersection of TVF and spatial filter, which leads to low detection performance in background clutter as shown in Figure 15(b). We solve these three problems by the hysteresis threshold-based detection after the temporal contrast filter (TCF). The TCF can enhance the signatures of moving target pixels and the hysteresis threshold-based detection can localize targets accurately.

**Figure 16:**
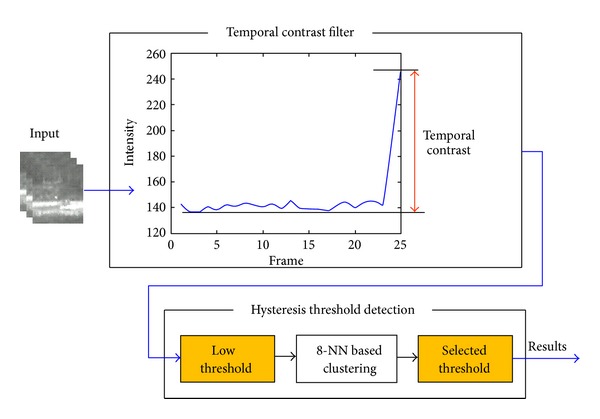
Proposed supersonic target detection system.

**Figure 17:**
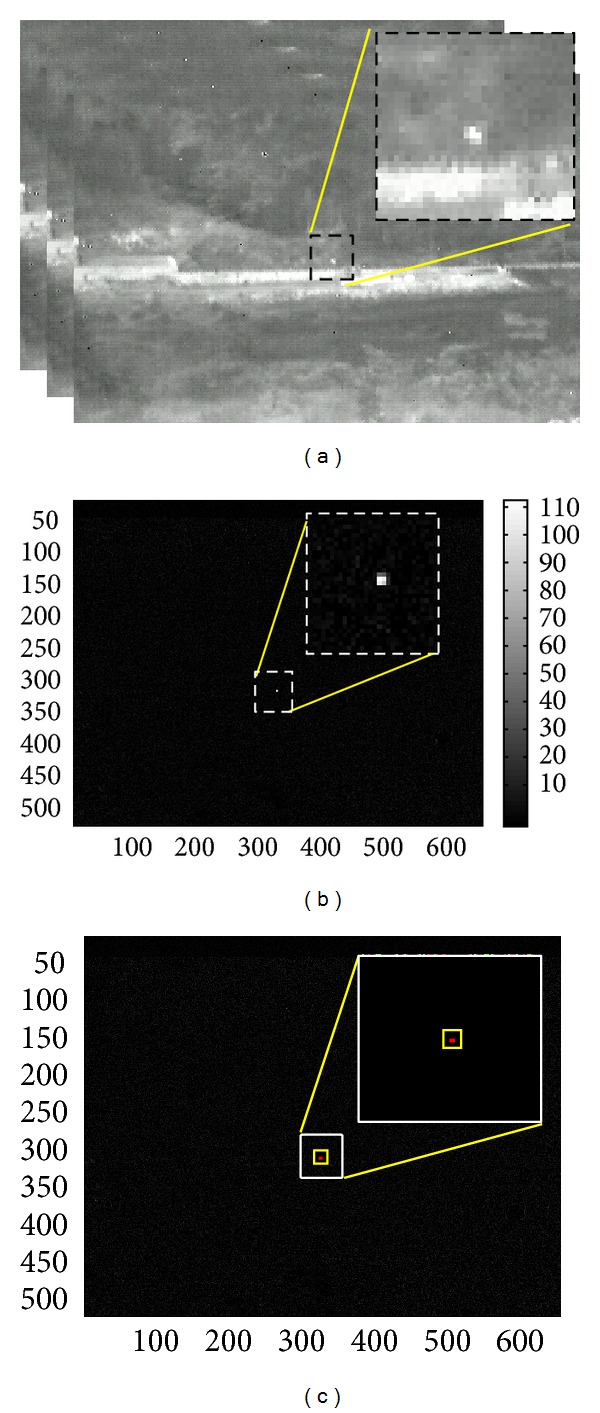
Supersonic target detection procedures with results: (a) input sequence, (b) TCF results, and (c) hysteresis threshold detection results.

*TCF-Based Supersonic Target Detection System.* The proposed small target detection system consists of TCF part and detection part, as shown in Figure 16. The filtering part conducts the enhancement of target signature by applying the temporal contrast. If *I*(*i*, *j*, *k*) denotes the intensity of (*i*, *j*) pixel at the current *k*th frame, the TCF at (*i*, *j*, *k*) is defined as (12). We assume that the buffer size is *k* and *k* − 1 frames are used to estimate background intensity. The key part of the TCF is the background signature estimation by the minimum filter to maximize the signal-to-noise ratio. Because the contrast is produced by the difference of the current intensity and previous intensity of a pixel, we can remove the ambiguity of target location. The rest of the detection system consists of a hysteresis thresholding method. The first threshold is selected to be as low as possible in order to find the candidate target region. Then the 8-nearest neighbor (8-NN) based clustering method is utilized to group the detected pixels. So, we can divide a considering region into a target region and a background region. The adaptive threshold detection is conducted by using (13) where *T*_max⁡_ denotes the maximal TCF in a target region and *μ*_BG_, *σ*_BG_ represent the average and standard deviation of the background region, respectively. If the signal-to-clutter ratio (SCR_temp_) is larger than a predefined threshold *t*, the current considering region is declared as a detected target.  Figure 17 summarizes the overall detection flow. Note that a moving target is enhanced by the proposed TCF and detected by the hysteresis threshold

(12) $$\begin{array}{c} \text{TCF}\left(i,j,k\right)=I\left(i,j,k\right)-\underset{n=1,2,\ldots ,k-1}{\min}I\left(i,j,n\right), \end{array}$$

(13) $$\begin{array}{c} \text{SC}{\text{R}}_{\text{temp}}=\frac{T_{\max}-\mu_{\text{BG}}}{\sigma_{\text{BG}}}. \end{array}$$

## 5. Experimental Results

**Figure 18:**
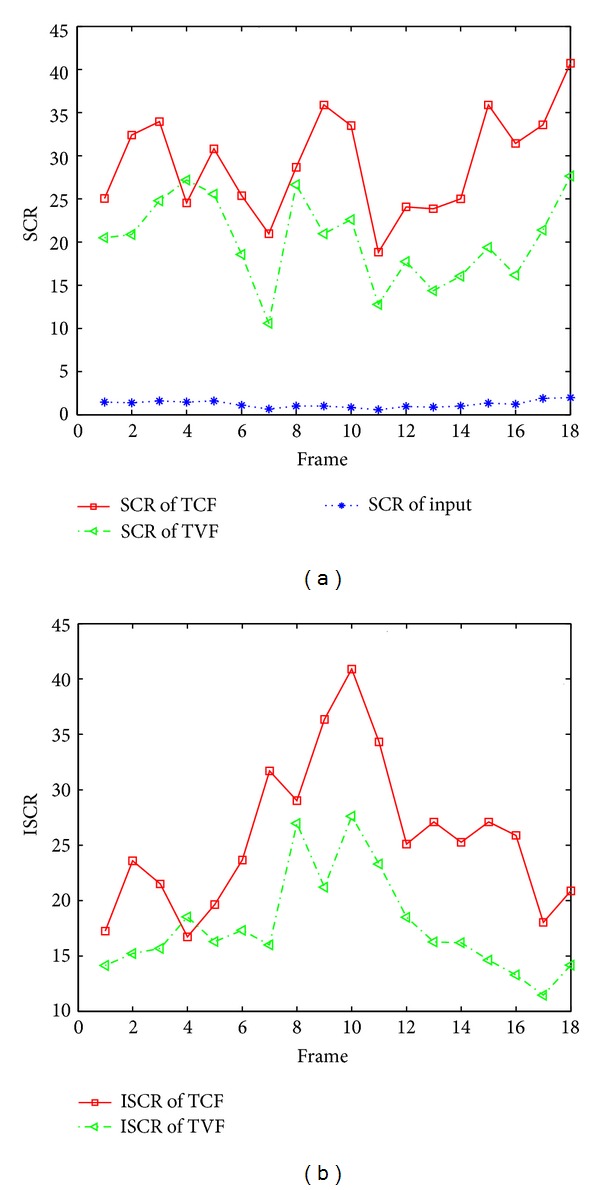
Comparative analysis of temporal filters for test sequence (Set 4): (a) SCR values for input, TCF, and TVF; (b) ISCR values for TCF and TVF.

**Figure 19:**
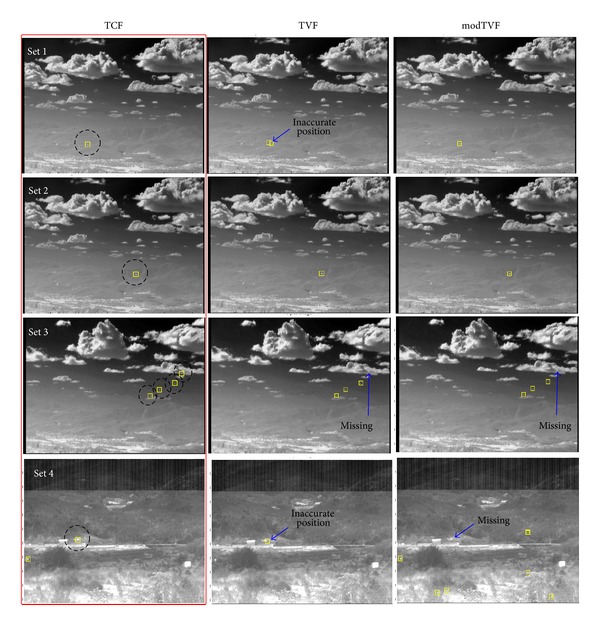
Performance comparison examples of small target detection methods for the four kinds of test sets. Circles represent ground truth and rectangles represent detected results.

We use the TVF as a baseline filtering method [8]. Targets are detected using the same hysteresis thresholding method. In addition, we compare the TCF with the modified TVF (modTVF) which uses both TVF and spatial filter (mean subtraction filter) to localize targets. We prepared two synthetic image sequences and two real target sequences (F-15, Metis-M) with frame rate of 120 Hz. The synthetic sequences are generated using the physics-based method [12]. A test target of Mach 3 is inserted in real ground clutter image with incoming path (Set 1) and passing-by path (Set 2). The first real sequence consists of four F-15s with dynamic motion in strong cloud clutter (Set 3). The second real sequence contains a real antitank missile (Metis-M) incoming near the IR camera (Set 4, Cedip, LWIR, 120 Hz). We evaluated the proposed method in terms of target detection performance as well as filtering performance. The filtering performance can be measured by the improvement of SCR (ISCR) that is defined as SCRout/SCRin. As shown in Figure 18, the proposed method outperforms the other in terms of ISCR for the test Set 4.  Table 5 summarizes the statistical performance comparisons of the proposed TCF, TVF, and modTVF in terms of detection rate and false alarm rate. We use the same temporal threshold (*t* = 7) and buffer size (*k* = 5) for fare comparisons. According to the results, the proposed temporal filter produces a much higher number of correct detections and lower localization errors than those of other methods.  Figure 19 shows small moving target detection results of cluttered images where the small rectangles represent detection. As indicated by the arrows, the TVF showed inaccurate target localizations due to the stripe patterns and the modTVF often missed true targets in clutter such as cloud edge and ground. Note the superior detection performance of the TCF-based method. The supplementary material on the web shows the demo sequences of target detection in Set3.

## 6. Conclusions

An analysis of the effects of environment in IR-based small target detection is very important. In this study, the 24-hour IR data was recorded in winter and spring, and the IR variations were analyzed in terms of the small target detection parameters particularly the signal-to-clutter ratio (SCR), which is the first trial in this area. SCR variations were analyzed with regard to the recording time, temperature, and humidity. According to the analysis, the natural backgrounds, such as mountains and near field, behave similarly. The SCR values increased during the recording time (10 H–09 H) in these regions. In addition, the SCR values decreased with increasing temperature and humidity. The SCR values of the sky background were quite high and did not show a specific pattern but were affected strongly by cloud. The SCR values of the man-made background, such as buildings, were almost constant regardless of the recording time, temperature, and humidity except spring. Overall, the best conditions can be determined for optimal small target detection or for predicting the small target detection performance under different weather conditions and backgrounds.

In terms of optimal target detection, IR signature variations should be considered to obtain desirable target detection rate and false alarm rate. If the background-related SCR variations are used, the small target detection system can be upgraded by controlling the detection thresholds adaptively depending on the background and weather conditions. However, such approach is impractical because it requires huge number of IR databases according to environmental parameters. On the other hand, we can overcome the IR variation by proposing a robust method. This paper proposed a new simple but powerful supersonic small target detection method by the novel temporal contrast filter. As validated by a set of experiments, it can effectively find and localize true targets with the velocity from subpixel to pixel per frame for various clutter images including cloud and ground clutter. Due to the simplicity of the algorithm with powerful detection capability, the proposed method can be used for real-time military applications for staring infrared cameras.

## Supplementary Material

The Set 3 sequence consists of four F-15s with dynamic motion in strong cloud clutter. The proposed TCF outperforms other TVF or TVFspatial in terms of target detection rate and localization accuracy.

## Figures and Tables

**Table 1:** Measurement sensors used during 24-hour recording.

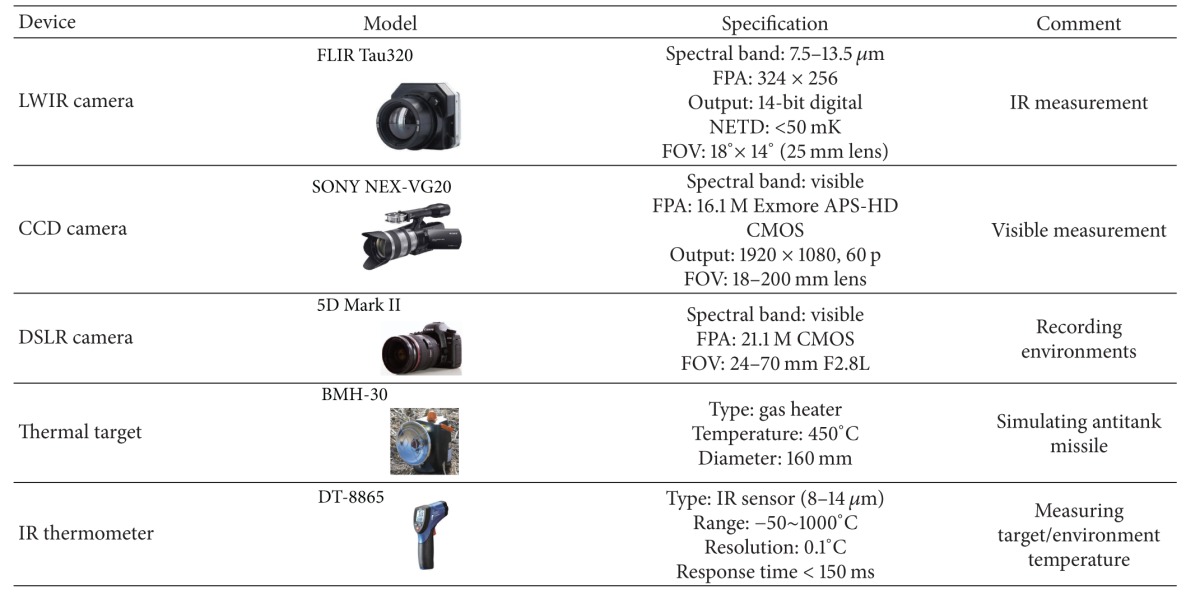

**Table 2:** Measured meteorological data of the recording place (winter).

Time	Weather	Visibility (km)	Cloud (1/10)	Temperature (°C)	Humidity (%)
2013.01.18.10 H	Clear	11	0	−2.3	45
2013.01.18.11 H	Clear	11	0	−0.6	31
2013.01.18.12 H	Clear	15	0	1.5	25
2013.01.18.13 H	Clear	15	0	2.8	18
2013.01.18.14 H	Clear	18	0	3.9	15
2013.01.18.15 H	Clear	20	0	4.8	14
2013.01.18.16 H	Clear	20	0	4.6	14
2013.01.18.17 H	Clear	20	0	3.6	16
2013.01.18.18 H	Clear	18	0	2.2	19
2013.01.18.19 H	Clear	20	0	1.4	22
2013.01.18.20 H	Clear	20	0	0.6	26
2013.01.18.21 H	Clear	20	0	0.3	27
2013.01.18.22 H	Clear	20	0	−0.2	32
2013.01.18.23 H	Clear	20	0	−1.2	47
2013.01.19.00 H	Clear	20	0	−1.2	52
2013.01.19.01 H	Clear	20	0	−1.4	56
2013.01.19.02 H	Clear	20	0	−1.5	56
2013.01.19.03 H	Clear	13	0	−2.1	60
2013.01.19.04 H	Clear	13	0	−2.3	61
2013.01.19.05 H	Clear	11	0	−2.5	63
2013.01.19.06 H	Clear	11	0	−2.5	62
2013.01.19.07 H	Cloudy	11	4	−2.9	65
2013.01.19.08 H	Cloudy	15	7	−3.0	66
2013.01.19.09 H	Cloudy	18	8	−2.6	65

**Table 3:** Measured meteorological data of the recording place (spring).

Time	Weather	Visibility (km)	Cloud (1/10)	Temperature (°C)	Humidity (%)
2013.04.19.11 H	Cloudy	23	5	12.8	32
2013.04.19.12 H	Cloudy	23	3	14.9	29
2013.04.19.13 H	Cloudy	23	5	14.5	28
2013.04.19.14 H	Clear	25	2	15.2	28
2013.04.19.15 H	Clear	25	2	15.4	27
2013.04.19.16 H	Clear	25	2	14.8	28
2013.04.19.17 H	Clear	25	4	13.5	30
2013.04.19.18 H	Cloudy	25	6	12.0	34
2013.04.19.19 H	Cloudy	23	6	10.3	39
2013.04.19.20 H	Cloudy	23	6	9.5	41
2013.04.19.21 H	Cloudy	20	6	9.1	43
2013.04.19.22 H	Cloudy	20	7	8.8	44
2013.04.19.23 H	Cloudy	20	7	8.8	44
2013.04.20.00 H	Cloudy	20	7	8.8	44
2013.04.20.01 H	Cloudy	20	7	8.8	43
2013.04.20.02 H	Cloudy	20	7	8.7	43
2013.04.20.03 H	Cloudy	20	10	8.6	46
2013.04.20.04 H	Cloudy	20	10	8.5	47
2013.04.20.05 H	Cloudy	20	10	8.5	48
2013.04.20.06 H	Cloudy	20	10	8.6	48
2013.04.20.07 H	Cloudy	20	10	8.5	50
2013.04.20.08 H	Cloudy	20	10	7.5	60
2013.04.20.09 H	Rainy	9	10	5.6	75

**Table 4:** Summary of the SCR variations according to the time, temperature, and humidity for various backgrounds.

Background	Time (10 H-09 H)	Temperature	Humidity
Mountain	Increase	Decrease	Increase
Near field	Increase	Decrease	Increase
Sky	High, fluctuating	High, fluctuating	High, fluctuating
Building (winter)	Constant	Constant	Constant
Building (spring)	Increase	Decrease	Increase

**Table 5:** Statistical performance comparisons of small infrared target detection methods (DR: detection rate, FAR: number of false alarms per frame, PE: position error).

Method	Performance measure	Set 1: syn. incoming	Set 2: syn. passing by	Set 3: real F-15 Multi	Set 4: real metis-M
TCF	DR (%)	100 (100/100)	100 (11/11)	98.78 (3,161/3,200)	99.13 (114/115)
	FAR (number/frame)	0 (0/100)	0 (0/11)	0.0025 (2/811)	0.4 (46/115)
	PE (pixel)	0.13	0.12	0.15	0.116

TVF	DR (%)	97 (97/100)	100 (11/11)	92.19 (2,950/3,200)	89.57 (103/115)
	FAR (number/frame)	0.6 (60/100)	4 (44/11)	0 (0/811)	0.16 (18/115)
	PE (pixel)	5.61	0.28	4.85	4.33

modTVF	DR (%)	100 (100/100)	100 (11/11)	91.09 (2,915/3,200)	36.52 (42/115)
	FAR (number/frame)	0.69 (69/100)	0 (0/11)	0.011 (9/811)	1.91 (220/115)
	PE (pixel)	0.15	0.14	0.17	0.17
